# Supplementary material for: High thermal conductivity in wafer-scale cubic silicon carbide crystals
Source: Nat Commun. 2022 Nov 23;13:7201. doi: 10.1038/s41467-022-34943-w (PMC9684120; doi:10.1038/s41467-022-34943-w)
Supplement: Supplementary file 1 — Supplementary Information [file 41467_2022_34943_MOESM1_ESM.pdf]

Supplementary Information

High Thermal Conductivity in Wafer-Scale Cubic Silicon Carbide Crystals

Zhe Cheng[1,*], Jianbo Liang[2,*], Keisuke Kawamura[3], Hao Zhou[4], Hidetoshi Asamura[5], Hiroki Uratani[3], Janak Tiwari[4], Samuel Graham[6], Yutaka Ohno[7], Yasuyoshi Nagai[7], Tianli Feng[4], Naoteru Shigekawa[2], David G. Cahill[1,*]

[1] Department of Materials Science and Engineering and Materials Research Laboratory, University of Illinois at Urbana-Champaign, Urbana, IL 61801, United States.

[2] Department of Electronic Information Systems, Osaka Metropolitan University, Sugimoto 3-3-138, Sumiyoshi, Osaka 558-8585, Japan.

[3] SIC Division, Air Water Inc. 2290-1 Takibe, Toyoshina Azumino, Nagano 399-8204, Japan.

[4] Department of Mechanical Engineering, University of Utah, Salt Lake City, Utah 84112, USA.

[5] Specialty Materials Dept., Electronics Unit, Air Water Inc. 4007-3 Yamato, Azusagawa, Nagano 390-1701, Japan.

[6] George W. Woodruff School of Mechanical Engineering, Georgia Institute of Technology, Atlanta, GA 30332, United States.

[7] Institute for Materials Research, Tohoku University, 2145-2 Narita, Oarai, Ibaraki 311-1313, Japan.

[*]Corresponding authors: zcheng18@illinois.edu; liang@omu.ac.jp; d-cahill@illinois.edu.

Supplementary Note 1: EBSD measurements

The image quality (IQ) map and the crystal direction map are shown in Supplementary Fig. 1 (face close to Si substrate) and Supplementary Fig. 2 (growth face). The EBSD data in Supplementary Figs. 1 and 2 show that the 3C-SiC has single (111) crystal orientation over the entire scanned area (2.4 mm×0.8 mm) on both faces.

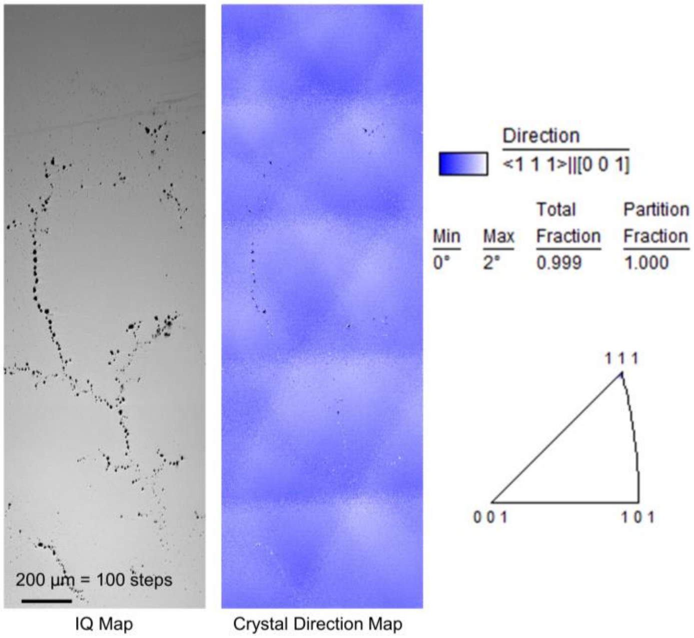

Supplementary Fig. 1. **EBSD map of 3C-SiC from the face close to Si substrate**. IQ map is the image quality map. The crystal direction map shows the spatial extent and amplitude of deviations of the orientations of the crystal (about 2 degree).

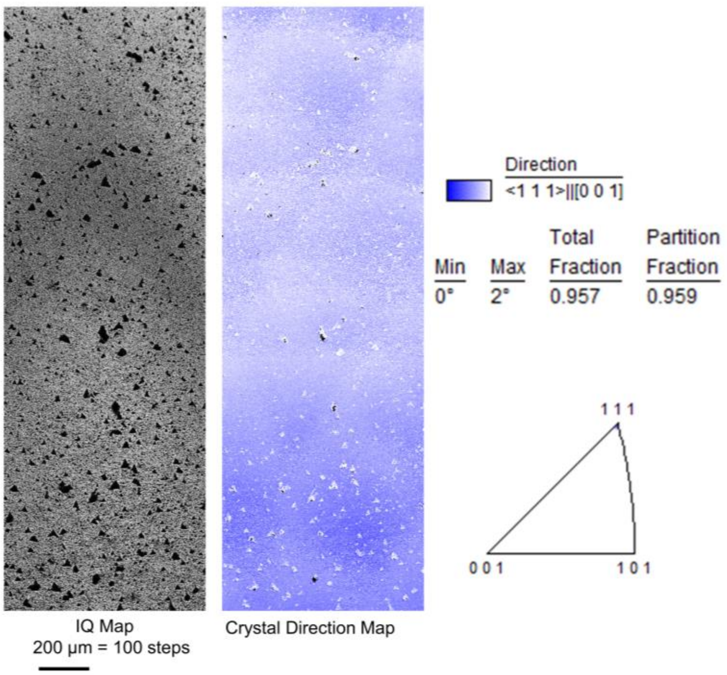

Supplementary Fig. 2. **EBSD map of 3C-SiC from the growth face**. IQ map is the image quality map. The crystal direction map shows the spatial extent and amplitude of deviations of the orientations of the crystal (about 2 degree).

Supplementary Note 2: SIMS data

The detailed second ion mass spectroscopy (SIMS) data is shown in Supplementary Fig. 3. The concentrations of O, N, and B are measured a function of depth from the surface.

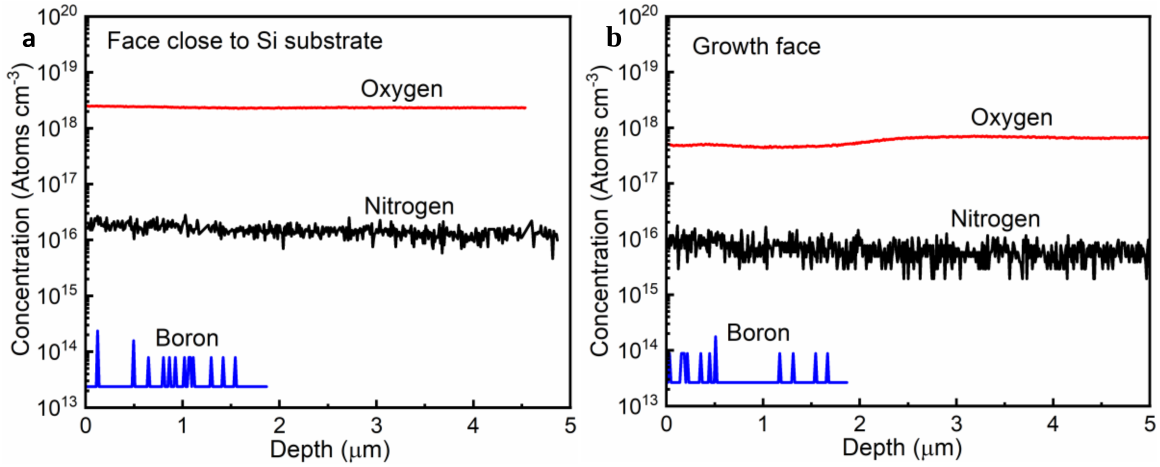

Supplementary Fig. 3. SIMS data of O, N, and B impurities. **a** data measured from the bottom side (close to the Si substrate). **b** data measured from the top side (growth side).

Supplementary Note 3: cross-plane κ of 3C-SiC, 4H-SiC, and 6H-SiC

Supplementary Fig. 4 shows the comparison of the temperature dependent cross-plane κ of 3C-SiC, 4H-SiC, and 6H-SiC bulk crystals.[1] The thermal conductivity of 3C-SiC is higher than those of the 4H-SiC and 6H-SiC at all the reported temperatures.

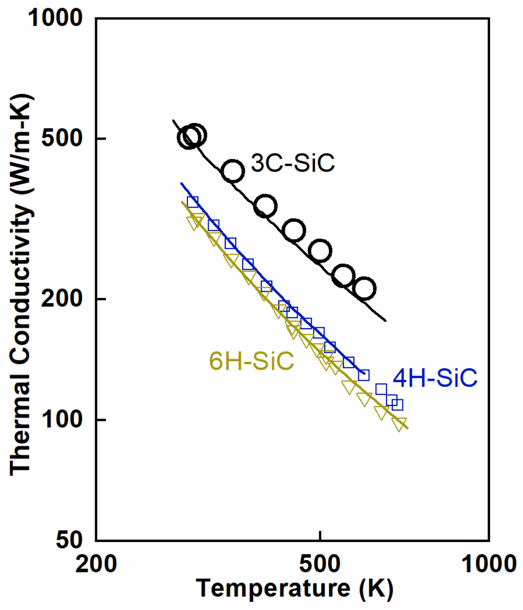

Supplementary Fig. 4 Temperature dependent cross-plane κ of 3C-SiC, 4H-SiC,[1] and 6H-SiC[1] bulk crystals.

Supplementary Note 4: TDTR data fittings

The temperature dependent thermal conductivity and heat capacity of Si are from literature.[2,3] The heat capacity data of 3C-SiC are the DFT-calculated values from Materials Project (DOI: 10.17188/1282015). Supplementary Fig. 5 shows the comparison of volumetric heat capacity of 3C-SiC, 4H-SiC, and 6H-SiC.[1] The thicknesses of Al transducer and 3C-SiC films are determined by picosecond acoustic technique (see below for more details).[4,5] The Al thermal conductivity is determined by measuring its electrical conductivity and applying Wiedemann-Franz law (170 W $m^{-1}K^{-1}$). The error bars of typical TDTR measurements are about ±10%.

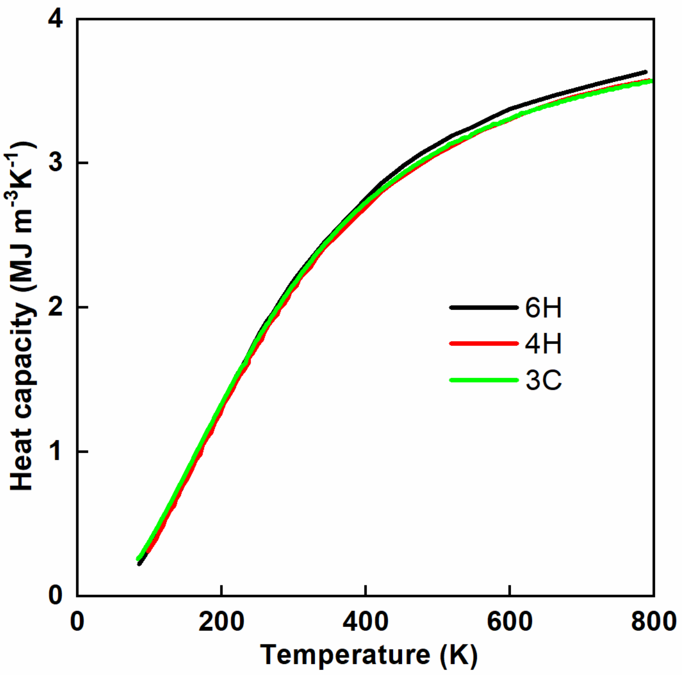

Supplementary Fig. 5 Temperature dependent heat capacity of 3C-SiC, 4H-SiC, and 6H-SiC.[1]

Supplementary Note 5: Picosecond acoustic technique

When the strain wave created by the pump laser reflects back from an interface, an echo shows up in the TDTR signal,[4] as shown in Fig. S6. According to the delay time the strain wave travels in the film, we are able to measure the thickness of that film with known sound velocity. The sound velocity of 3C-SiC along [111] direction is 12.5 km/s.[6] The sound velocity of Al is 6.4 km/s. The measured thicknesses of the 3C-SiC films are listed in Supplementary Fig. 6.

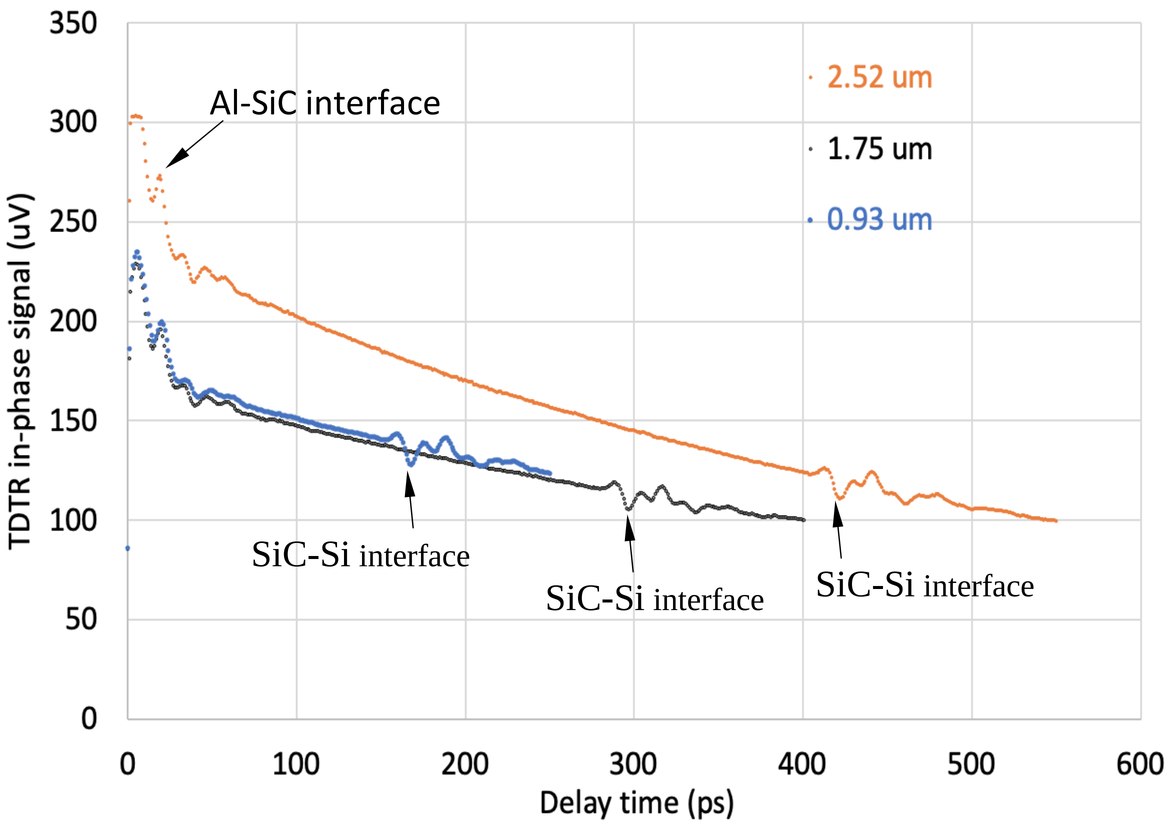

Supplementary Fig. 6 Picosecond acoustic echoes of the 3C-SiC on Si samples with different 3C-SiC film thicknesses. The measured thicknesses of the 3C-SiC films are listed in the figure.

Supplementary Note 6: Beam offset TDTR

The beam offset measurements were performed at a delay time of -50 ps with a 10× objective and a modulation frequency of 1.9 MHz. The measured full width at half maximum (FWHM) of the

out-of-phase TDTR signal as a function of beam offset distance in the beam offset experiments is fitted with the calculated FWHM values from analytical heat transport solution of the sample structure to obtain in-plane thermal conductivity of 3C-SiC films. Supplementary Fig. 7 shows an example of the data fitting process of a BO-TDTR measurement on the 2.52-μm-thick 3C-SiC thin films. The uncertainty of in-plane thermal conductivity due to the FWHM uncertainty is ±6 W $m^{-1}K^{-1}$, which is small compared to other errors in the experiment. The fitted in-plane thermal conductivity of the 3C-SiC film is 350±35 W $m^{-1}K^{-1}$. The sensitivity of the in-plane and cross-plane thermal conductivity of the 0.93-μm-thick 3C-SiC film is small so we report a ±20% error bar in this work.

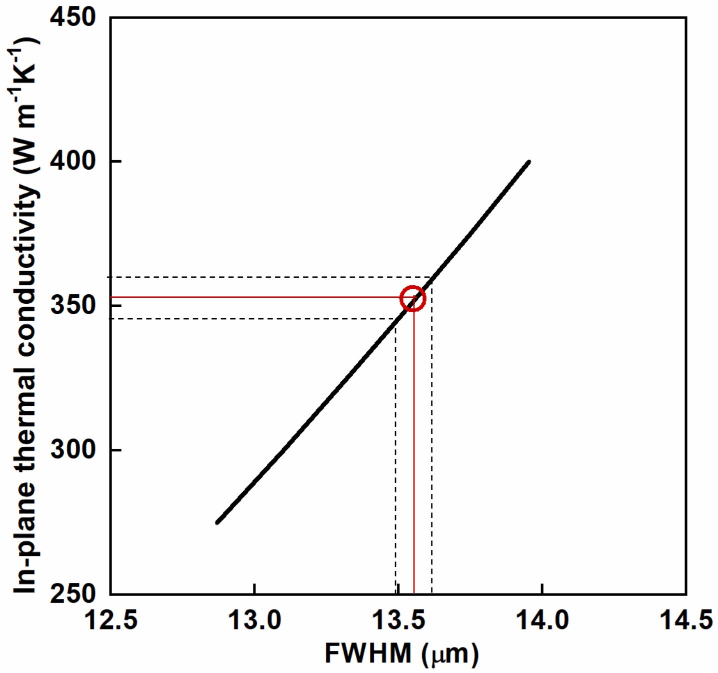

Supplementary Fig. 7 Data fitting of BO-TDTR for the 2.52-μm-thick 3C-SiC thin films. The red circle is the fitted in-plane thermal conductivity with the measured FWHM. The measured full width at half maximum (FWHM) is from the out-of-phase TDTR signal as a function of beam offset distance in the beam offset experiments. The black line is the simulated values according to

the sample structure. The black dash lines are the uncertainties in the measurements of in-plane thermal conductivity as a result of the FWHM uncertainties (0.05 μm).

Supplementary Note 7: TBC measurements

TDTR is performed on the 1.75 μm and 0.93 μm 3C-SiC on Si samples to measure the 3C-SiC-Si TBC. The data fittings of the TDTR ratio and fitted results are shown in Supplementary Fig. 8. Consistent 3C-SiC-Si TBC values are obtained in both measurements (~620 MW $m^{-2}K^{-1}$). Similar measurements are also performed on the 2.52 μm 3C-SiC on Si sample but it is insensitive to the 3C-SiC-Si TBC because of the large thickness.

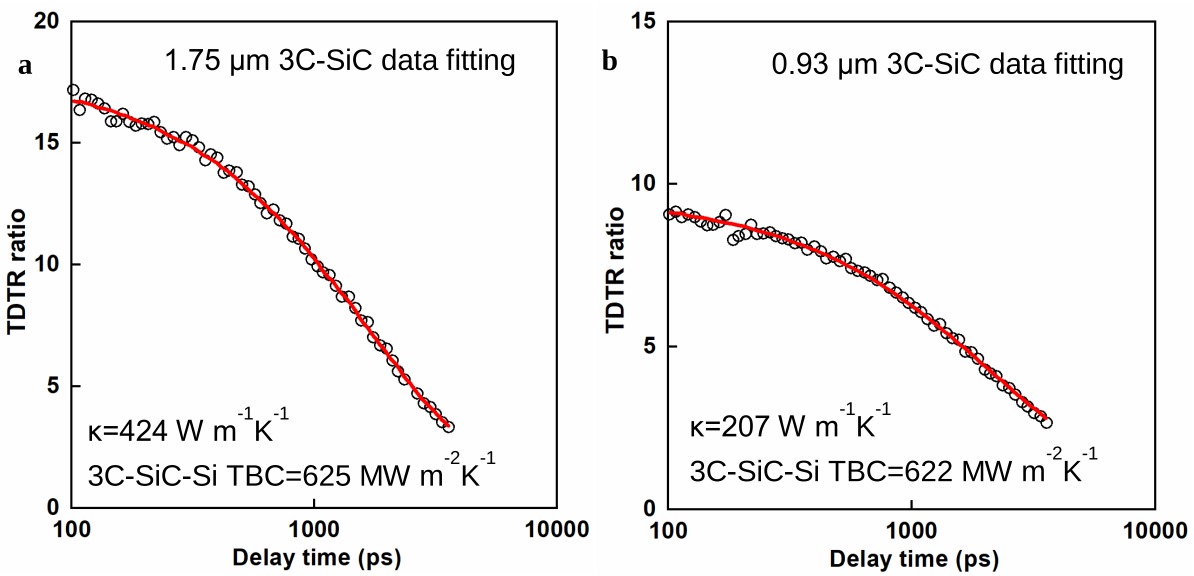

Supplementary Fig. 8 TDTR ratio fitting of the measurements. **a** 1.75 μm 3C-SiC on Si sample with 5× and 9.3 MHz. **b** 0.93 μm 3C-SiC on Si sample with 5× and 9.3 MHz. The circles are experimental data while the red lines are the fitting curves. The fitted results are also included.

To further check the fitted data in the Supplementary Fig. 8, we force the 3C-SiC-Si TBC as a certain value close to 620 MW m^{-2}K^{-1} and refit the κ of the 3C-SiC thin films. The relation between the 3C-SiC-Si TBC and the κ of the 3C-SiC thin films are shown in Supplementary Fig. 9. The empty circles are the first set of measuremnets while the filled circles are repeating data collected on different spots on corresponding samples. Due to size effect, thicker film has higher thermal conductivity. Here, 2.52-μm-thick 3C-SiC film should have higher κ than that of the 1.75-μm-thick 3C-SiC film. According to Supplementary Fig. 9, the 3C-SiC-Si TBC needs to be higher than 500 MW m^{-2}K^{-1}. The measured 3C-SiC-Si TBC (620 MW m^{-2}K^{-1}) agrees with the measurements on these three samples. Thus, the best-fit value of the 3C-SiC-Si TBC is 620 MW m^{-2}K^{-1} with a lower limit of 500 MW m^{-2}K^{-1}.

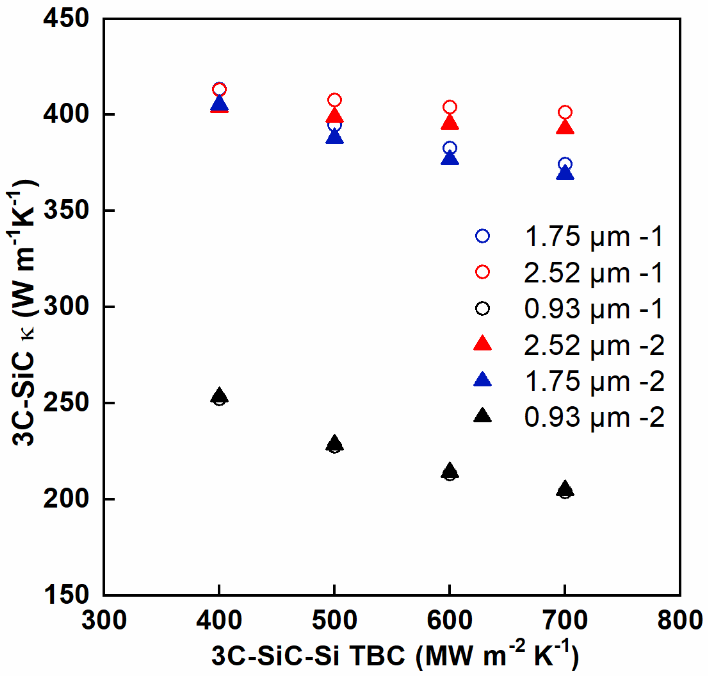

Supplementary Fig. 9. Refiting TDTR data of the three 3C-SiC on Si samples. The κ of the 3C-SiC thin films are fitted by forcing the 3C-SiC-Si TBC as a certain value. There are two sets of data points which are measured on different spots on the corresponding samples.

Supplementary Note 8: XRD FWHM

Supplementary Fig. 10 shows the full width at half maximum (FWHM) of SiC (111) peak in the rocking curve of X-ray diffraction on the 3C-SiC thin films as a function of film thickness grown on Si substrates. The data of NovaSiC and Matsunami are from literature.[7] The lower values of FWHM of our samples than literature values show the improved quality of 3C-SiC crystals. As the films grow thicker, the FWHM values decrease. The 3C-SiC near the 3C-SiC-Si interfaces have relatively low crystal quality while the quality improves as the films grow thicker.

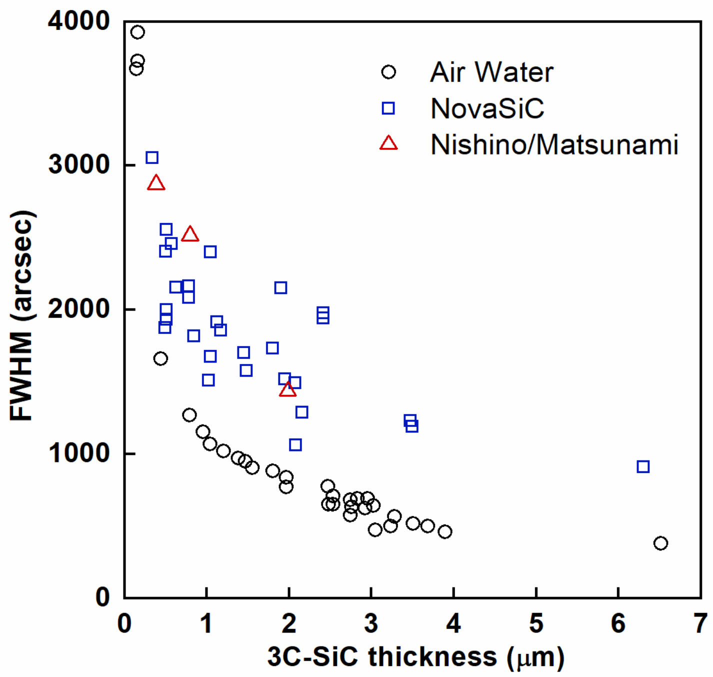

Supplementary Fig. 10 The full width at half maximum of SiC (111) peak in the rocking curve of X-ray diffraction on the 3C-SiC thin films as a function of film thickness grown on Si substrates. Air water is our sample source in this work. The data of NovaSiC and Matsunami are from literature.[7]

Supplementary Note 9: 3C-SiC-AlN interfaces

To measure the TBC of 3C-SiC-AlN interfaces, the sample was fabricated as shown in Supplementary Fig. 11. A layer of 1 μm 3C-SiC is grown on a Si substrate before epitaxial growth of AlN and GaN layers. Then the GaN is bonded to a Si template before etching away the Si substrate. After polishing the exposed 3C-SiC surface, a layer of Al is coated as TDTR transducer. The thickness of 3C-SiC layer is 887 nm and the AlN layer is 2530 nm. In TDTR measurements, only the Al layer, 3C-SiC layer, and the AlN layer are considered in the data fitting since AlN is thermally thick with a modulation frequency of 9.3 MHz. The thermal conductiviy of AlN layer used 200 W m^{-1} K^{-1} in the data fitting by refering to literature values of epitaxial AlN films.[8,9] The reported TBC value in the main text is the best-fit value.

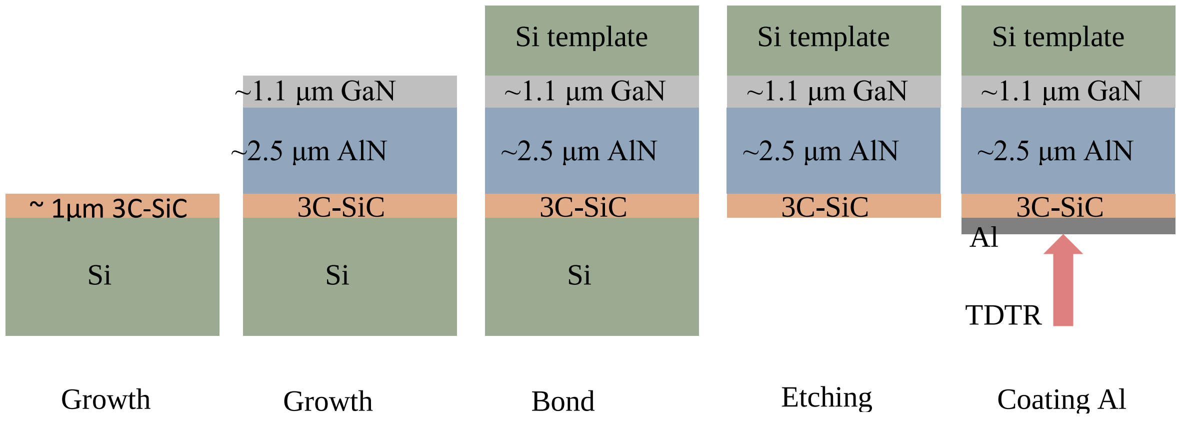

Supplementary Fig. 11. Sample fabrication for TBC measurements of 3C-SiC-AlN interfaces.

The STEM study of the 3C-SiC-AlN interfaces are shown in Supplementary Fig. 12. The FFT data of AlN is shown in Supplementary Fig.12a while the orientations and lattice constants of both AlN and 3C-SiC are shown in Supplementary Fig. 12b.

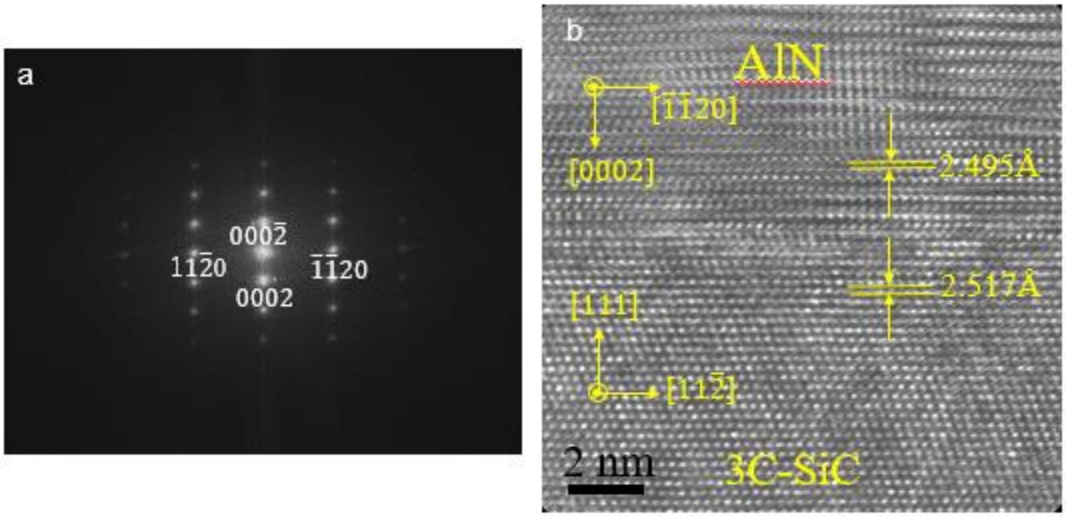

Supplementary Fig. 12 STEM study of the 3C-SiC-AlN interfaces. **a** Fast Fourier transform (FFT) of the STEM image of the AlN. **b** Orientations and lattice constants of AlN and 3C-SiC.

Supplementary Note 10: First principle calculations

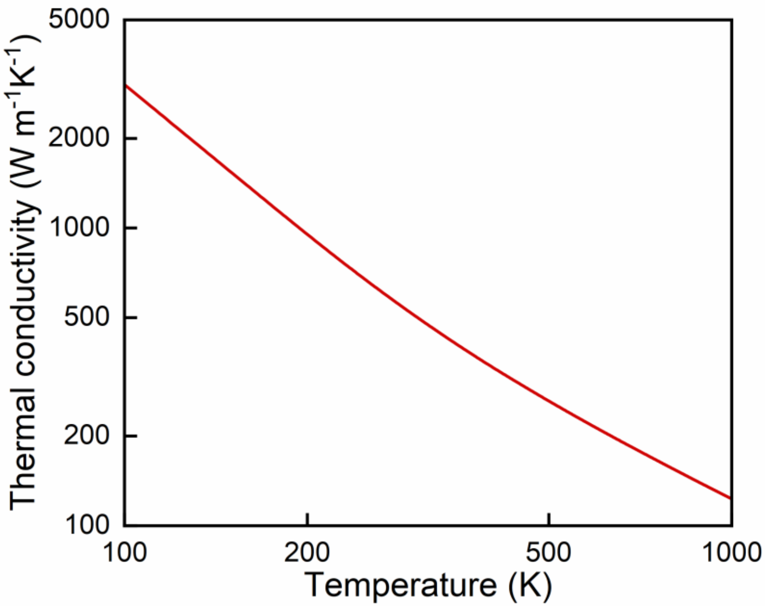

Supplementary Fig. 13. First principle calculated thermal conductivity of perfect single crystal 3C-SiC.

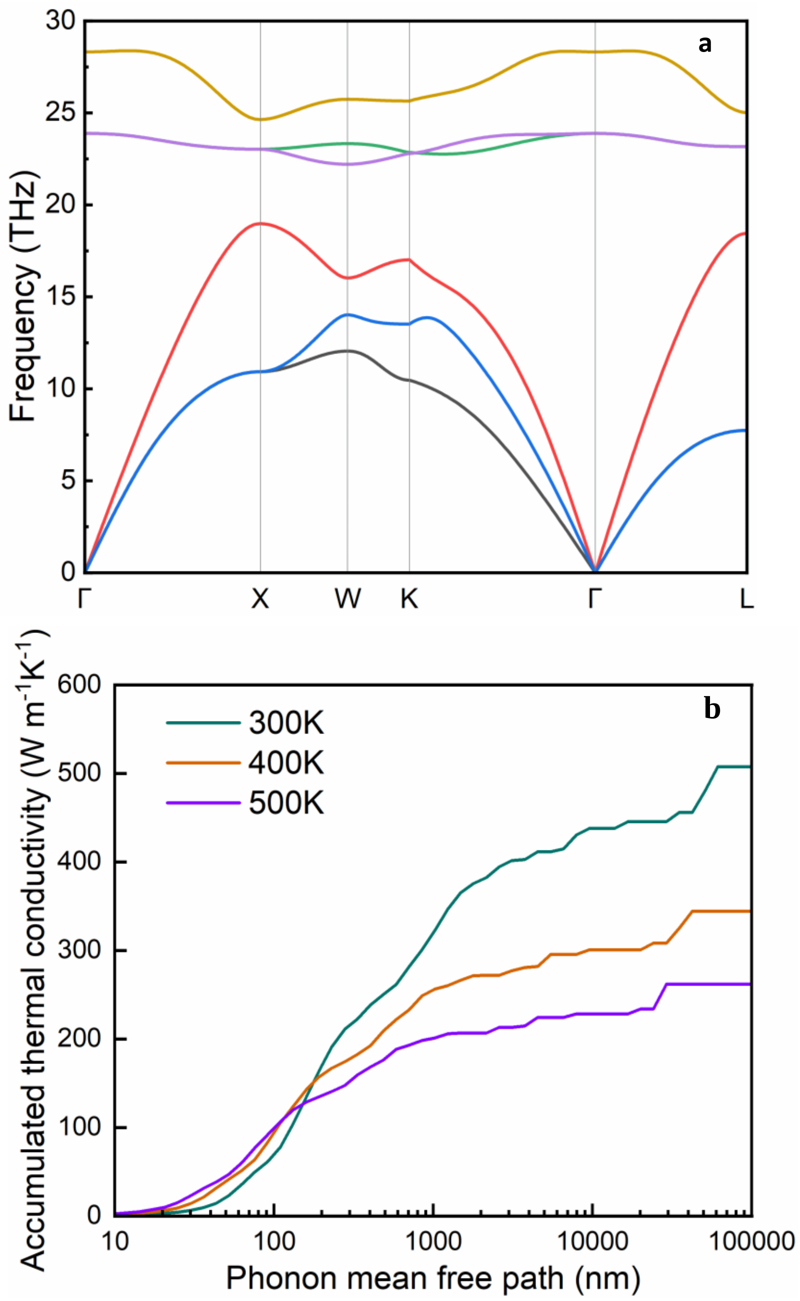

Supplementary Fig. 14. Calculated phonon properties of perfect 3C-SiC single crystal. **a** Phonon dispersion relation of perfect 3C-SiC single crystal. **b** Accumulated thermal conductivity of perfect 3C-SiC single crystal at 300, 400, and 500 K. It shows the contributions of phonons with different mean free paths to the total thermal conductivity.

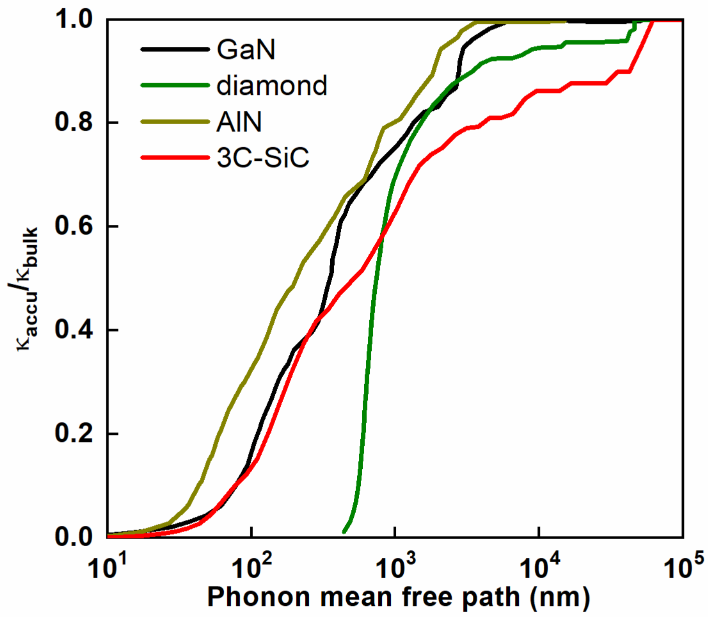

Supplementary Fig. 15. The accumulated thermal conductivity of 3C-SiC, AlN, GaN, and diamond scaled by their bulk thermal conductivity at room temperature.

Supplementary Note 11: Laser information

The Raman measurements and TDTR measurements use lasers. The laser information of the Raman system can be found in the product manual (Horiba LabRAM). The TDTR laser is a Spectra-Physics pulsed Ti-sapphire Tsunami laser. Detailed information can be found in the product manual.

Supplementary Table 1. The list of thermal conductivity of nanocrystalline diamond thin films and the corresponding references.

Thickness	k_in-plane	k_out	References
μm	W m^{-1} K^{-1}	W m^{-1} K^{-1}	
1.06	117		[10]
3.02	157		[10]
1.1	95	175	[11]
1	103		[12]
0.47	40		[13]
0.475	90		[13]
0.68	65		[13]
1	75		[13]
1	85		[13]
1	130		[13]
0.98	145		[13]
1.5	190		[13]
0.5	52		[14]
1	77	210	[14]
5.6	130	710	[14]
2		280	[15]
2		310	[15]
2		360	[15]
5		703	[16]
1	93	180	[17]

Supplementary References

1 Zheng, Q. *et al.* Thermal conductivity of GaN, GaN 71, and SiC from 150 K to 850 K. *Phys. Rev. Mater.* **3**, 014601 (2019).

2 Fulkerson, W., Moore, J., Williams, R., Graves, R. & McElroy, D. Thermal conductivity, electrical resistivity, and seebeck coefficient of silicon from 100 to 1300 K. *Phys. Rev.* **167**, 765 (1968).

3 Touloukion, Y. Thermophysical properties of matter. *IFI/PLENUM New York-Washington* **153** (1970).

4 Cheng, Z. *et al.* Thermal conductance across β-Ga2O3-diamond van der Waals heterogeneous interfaces. *APL Mater.* **7**, 031118 (2019).

5 Hohensee, G. T., Hsieh, W.-P., Losego, M. D. & Cahill, D. G. Interpreting picosecond acoustics in the case of low interface stiffness. *Rev. of Sci. Instrum.* **83**, 114902 (2012).

6 Davydov, S. Y. Effect of pressure on the elastic properties of silicon carbide. *Phys. of the Solid State* **46**, 1200-1205 (2004).

7 Cordier, Y. *et al.* AlGaN/GaN high electron mobility transistors grown on 3C-SiC/Si (1 1 1). *J. of Crystal Growth* **310**, 4417-4423 (2008).

8 Hoque, M. S. B. *et al.* High in-plane thermal conductivity of aluminum nitride thin films. *ACS Nano* **15**, 9588-9599 (2021).

9 Alvarez-Escalante, G. *et al.* High thermal conductivity and ultrahigh thermal boundary conductance of homoepitaxial AlN thin films. *APL Mater.* **10**, 011115 (2022).

10 Hines, N. J. *et al.* Steady-state methods for measuring in-plane thermal conductivity of thin films for heat spreading applications. *Rev. of Sci. Instrum.* **92**, 044907 (2021).

11 Anaya, J. *et al.* Simultaneous determination of the lattice thermal conductivity and grain/grain thermal resistance in polycrystalline diamond. *Acta Materialia* **139**, 215-225, (2017).

12 Yates, L. *et al.* Simultaneous Evaluation of Heat Capacity and In-plane Thermal Conductivity of Nanocrystalline Diamond Thin Films. *Nano. and Micro. Thermophys. Eng.* **25**, 166-178 (2021).

13 Anaya, J. *et al.* Control of the in-plane thermal conductivity of ultra-thin nanocrystalline diamond films through the grain and grain boundary properties. *Acta Materialia* **103**, 141-152 (2016).

14 Sood, A. *et al.* Anisotropic and inhomogeneous thermal conduction in suspended thin-film polycrystalline diamond. *J. of Appl. Phys.* **119**, 175103 (2016).

15 Cheng, Z. *et al.* Tunable Thermal Energy Transport across Diamond Membranes and Diamond-Si Interfaces by Nanoscale Graphoepitaxy. *ACS Appl. Mater. & Interf.* **11** (20), 18517-18527, (2019).

16 Yates, L. *et al.* Characterizations of the thermal conductivity of CVD diamond for GaN-on-diamond devices, *Compound Semiconductor Integrated Circuit Symposium (CSICS), IEEE.* 1-4 (2016).

17 Cheaito, R. *et al.* Thermal conductivity measurements on suspended diamond membranes using picosecond and femtosecond time-domain thermoreflectance, *Thermal and Thermomechanical Phenomena in Electronic Systems (ITherm), 16th IEEE Intersociety Conference on.* 706-710, (2017).
